# Supplementary material for: Enhancing the discriminatory power of polygenic scores for ADHD and autism in clinical and non-clinical samples
Source: J Neurodev Disord. 2025 Jun 9;17:32. doi: 10.1186/s11689-025-09620-w (PMC12150501; doi:10.1186/s11689-025-09620-w)
Supplement: Supplementary file 2 — Supplementary Material 2. [file 11689_2025_9620_MOESM2_ESM.docx]

Supplemental Figures


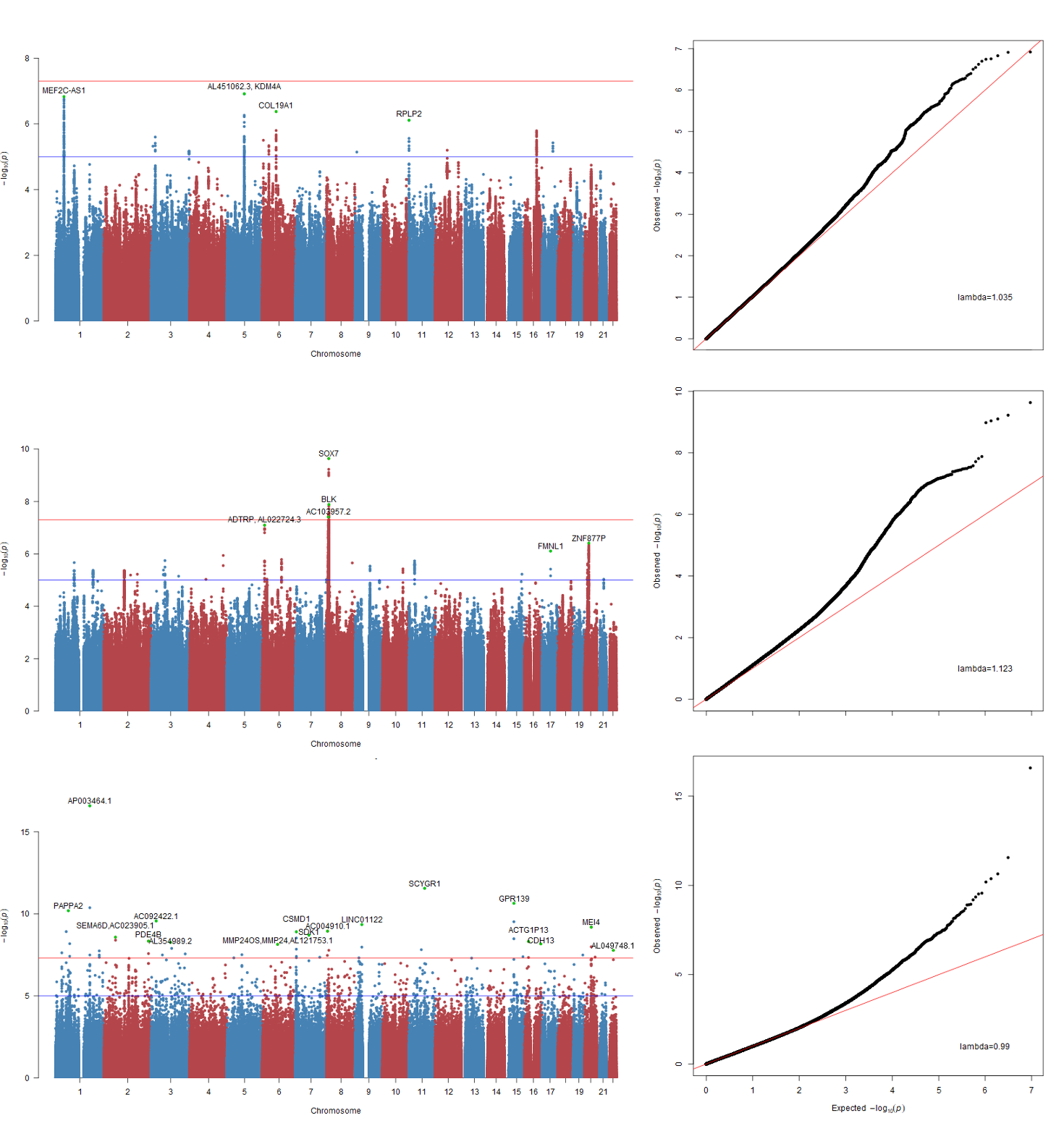


**Figure S1.** The Manhattan and QQ plots for GenomicSEM ADHD (top panels), GenomicSEM ASD (middle panels) and Neurodevelopmental GWAS (bottom panels).

**
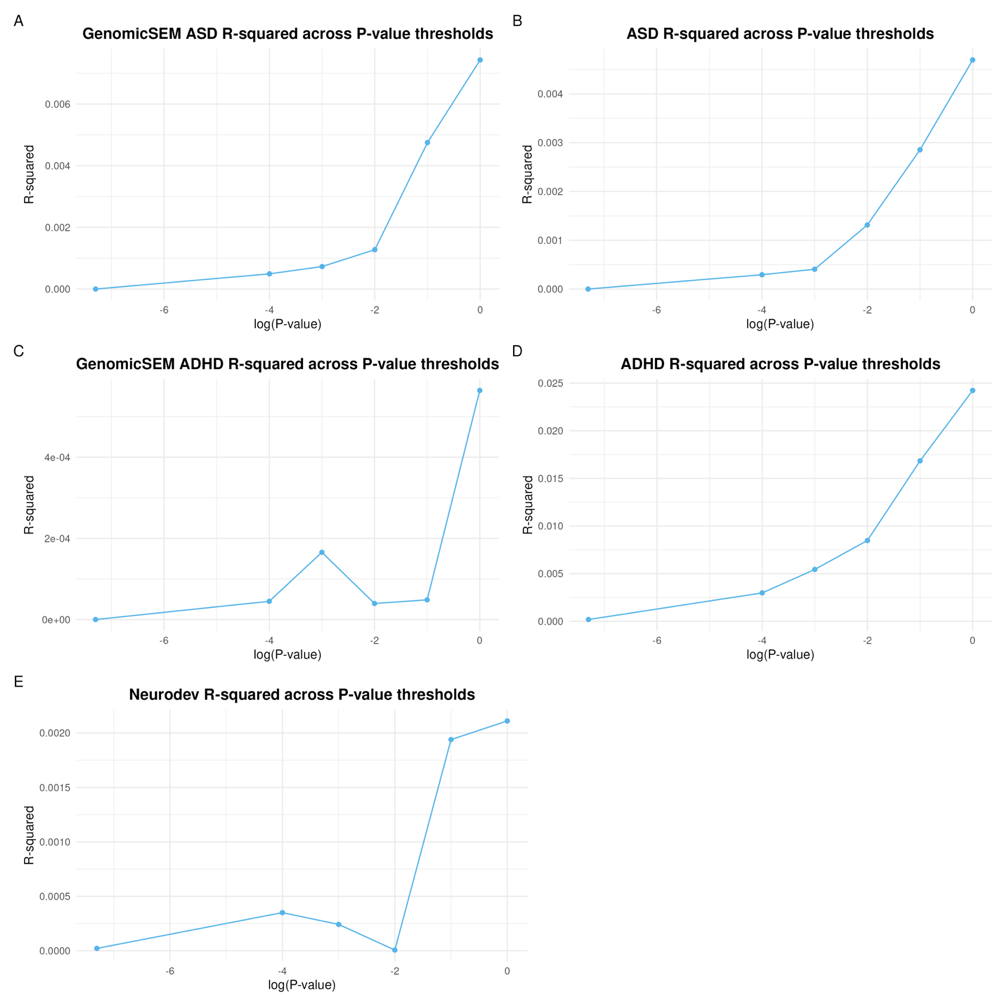
Figure S2**. PGS variance explained (*r*^2^) as a function of *p*-value thresholds of 1, 0.1, 0.01, 0.001, 0.0001, and 5 x 10^-8^ using PUMAS optimization method; each input GWAS (i.e., traditional ADHD GWAS, traditional ASD GWAS, GenomicSEM ADHD GWAS, GenomicSEM ASD GWAS and the Neurodevelopment GWAS) was split into training (75% of total data) and test (25% of total data) splits with 4 folds of cross-validation.
